# Prevalence of Anterior Femoral Neck Osteophyte in a Total Hip Arthroplasty Population: Analysis of Preoperative Surgical Plans

**DOI:** 10.1155/2019/5193945

**Published:** 2019-03-03

**Authors:** Adam M. Katchky, Mitchell L. Smith, Andrew J. Shimmin, Stephen J. McMahon, Jeremy Latham, Jonathan V. Baré

**Affiliations:** ^1^Department of Orthopedics and Rehabilitation, University of Vermont, Burlington, VT, USA; ^2^Melbourne Orthopaedic Group, Windsor, VIC, Australia; ^3^Department of Surgery, Monash University, Melbourne, VIC, Australia; ^4^Malabar Orthopaedic Clinic, Windsor, VIC, Australia; ^5^Spire Southampton Hospital, Southampton, UK

## Abstract

Despite strongly positive results of total hip arthroplasty (THA), patients remain at risk for complications including dislocation. Spinopelvic motion and the hip-spine relationship have been recognized as important factors in surgical planning and implant positioning in THA. Periarticular osteophytes are one of the hallmark pathoanatomic features of osteoarthritis and may influence implant positioning and joint stability; residual osteophytes at the anterior femoral neck may cause anterior impingement and posterior instability. No studies have been identified which establish the prevalence of anterior femoral neck osteophyte for incorporation into THA planning. 413 consecutive patients scheduled for THA underwent preoperative planning taking into account spinopelvic motion to establish optimal component position. Each surgical plan was reviewed retrospectively by four independent raters who were blinded to other imaging and intraoperative findings. Anterior femoral neck osteophytes were rated as being absent, minor, or extensive for each case. A single outlying rater was excluded. Inter-rater reliability was calculated manually. The patient group comprised 197 male and 216 female hips, with a mean age of 63 years (range 32–91). The presence of anterior femoral neck osteophytes was identified in a mean of 82% of cases (range 78–86%). A significant number of patients were found to have large or extensive osteophytes present in this location (mean 27%; range 23–31%). Inter-rater reliability was 70%. A large majority of our THA patients were found to have anterior femoral neck osteophytes. These must be considered during preoperative planning with respect to the spinopelvic relationship. Failure to identify and address osteophytes intraoperatively may increase the risk of impingement in flexion and/or internal rotation, leading to decreased range of motion, joint instability, and possibly dislocation. Planned future directions include incorporation of an impingement and instability model into preoperative planning for THA.

## 1. Introduction

Total hip arthroplasty (THA) is a common surgical procedure to treat osteoarthritis (OA) and other hip conditions, with over 91,000 cases reported in the UK in 2017 [1]. Despite significant advances in surgical technique and implant design, patients remain at risk of joint instability and dislocation following THA. Estimates for the rate of dislocation following primary THA have ranged from 0.3 to 10% [2–6]. Numerous patient, surgical, and implant factors may augment the risk of dislocation [2, 3, 6]. Impingement is defined as the abnormal contact between the femoral and acetabular sides during physiologic hip range of motion. Among the multiple causes of dislocation following THA, impingement may be a significant contributing factor [7]. THA impingement may result from contact of the implant, bony, or soft tissue structures. In addition to dislocation, THA impingement may lead to restricted range of motion, subluxation, edge loading, accelerated wear, and increased pain [7]. Impingement may occur at any location around the acetabulum, most commonly at the anterior or posterior walls. Anterior impingement following THA is often caused by residual anterior osteophyte on the femoral or acetabular side. Anterior impingement tends to cause increased edge loading and asymmetric wear at the posterior aspect of the acetabulum, as well as posterior subluxation or dislocation.

Implant positioning is critically important to ensure THA stability [2, 7]. The landmark paper by Lewinnek [8] established the gold standard “safe zone” for acetabular component positioning as 40°  ±  10° abduction (inclination) and 15°  ±  10° anteversion. However, even the Lewinnek cohort included patients within the safe zone who suffered THA dislocation, and other papers have further questioned the presence and location of a true “safe zone” to prevent dislocation [9]. One explanation for this may lie in the spinopelvic relationship. The pelvis is known to tilt forward and backward (flex/extend) with activities of daily living, including climbing stairs, lying, sitting, or rising from a seated position [10]. Further, considerable variation has been demonstrated between individuals including the pattern, direction, and degree of pelvic motion [10]. Differences in spinopelvic motion may significantly impact the instantaneous functional position of a THA acetabular component [11, 12]. For example, anterior rotation (flexion) of the pelvis will lead to an effectively more flat and retroverted cup position, increasing the risk of anterior impingement and posterior instability.

In view of this evolving understanding of the impact of the spinopelvic relationship on THA, numerous techniques and technologies have been developed which accommodate for pelvic motion in the placement of THA implants. One such technology is the Optimized Positioning System (OPS™, Corin Group, Gloucestershire, UK). This system utilizes preoperative lateral pelvic radiographs in multiple functional positions, as well as a low-dose CT scan, to assess patient-specific anatomy and spinopelvic motion and guide THA positioning to maximize range of motion and minimize impingement [13]. A surgical plan is developed for each patient, including 2D imaging and 3D reconstructions of patient anatomy, and planned implant positions.

In spite of the importance of bony anatomy and implant positioning to the stability of THA, no studies have been identified which assess the presence and size of anterior femoral neck osteophytes in patients undergoing THA. The identification of osteophytes in this area may be particularly important in patients with certain patterns of spinopelvic motion, specifically those who display increased pelvic flexion when sitting. This study aimed to use presurgical templating documents to establish the prevalence of anterior femoral neck osteophytes for incorporation into THA planning.

## 2. Materials and Methods

**Figure 1:**
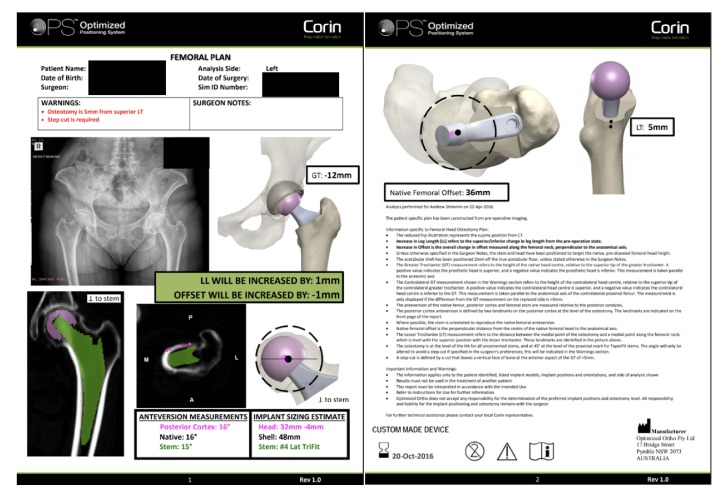
Case example of a presurgical plan developed using the OPS™ system, analyzed to assess the presence and size of anterior femoral neck osteophytes.

Surgical plans were retrospectively reviewed for all patients who underwent primary THA using the OPS™ system at a single hospital in Australia between November, 2015, and December, 2016. All patients had previously undergone standardized preoperative assessment including routine radiographs of the pelvis and operative hip, lateral pelvic radiographs in 3 stances (standing, seated, and contralateral leg step-up), and low-dose computerized tomography (CT) scan of the operative hip. Preoperative surgical plans were generated using the OPS™ system, taking into account spinopelvic motion in order to establish the optimal component positions to minimize impingement and maximize range of motion and stability. Each surgical plan includes an AP pelvic radiograph, two CT cuts (coronal plane, central implant axis; axial plane, level of planned femoral neck cut), and three 3D reconstruction images of the proximal femur (coronal, sagittal, and axial views) with the planned femoral implant in situ. An example of the surgical plan generated is shown in Figure 1.

**Figure 2:**
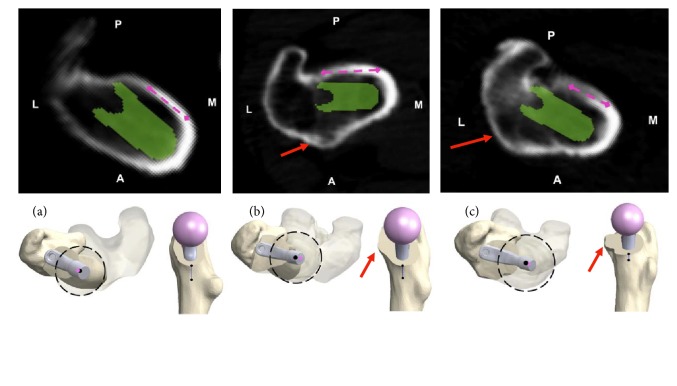
Case examples showing axial CT scan slices in the plane of the planned femoral neck osteotomy, with corresponding 3D reconstructions in the axial and sagittal planes (red arrows indicate anterior femoral neck osteophyte): (a) no anterior femoral neck osteophyte; (b) minor osteophyte; (c) extensive osteophyte.

Each planning document was reviewed by four independent assessors (one orthopaedic registrar, one fellow in lower extremity reconstruction, and two fellowship trained arthroplasty surgeons). Assessors were permitted to inspect all images contained within the plan, in the order of their choosing. Assessors were blinded to patient identity, any other imaging which may have been conducted, and any intraoperative findings. Each assessor examined each case to assess the presence and size of osteophytes at the anterior aspect of the femoral neck. In each case, osteophyte was judged to be absent (no bone extending beyond anterior cortex), minor (osteophyte encompassing ≤50% of anterior neck and extending ≤ 5 mm), or extensive (encompassing >50% of anterior neck and/or extending >5 mm). Examples of cases with no, minor, and extensive anterior neck osteophytes are displayed in Figure 2. Scores from each of the four assessors were compiled for each patient case. Descriptive statistics and inter-rater reliability were calculated manually in Apple Numbers [version 5.3 (5989)]. A single assessor was found to be significantly divergent from the other three. Analyses were repeated with all four assessors included and again with the single outlying assessor excluded.

## 3. Results and Discussion

### 3.1. Patient Demographics

A total of 413 cases were identified with surgical planning documents available. No patient cases were excluded. The group comprised 197 (48%) male hips and 216 (52%) female hips. Mean age of patients at the time of surgery was 63 years (SD = 10, range 32–91 years). 227 cases (55%) were planned for right THA, compared to 186 cases (45%) on the left hip. Descriptive data on the patient cohort is summarized in Table 1.

### 3.2. Anterior Femoral Neck Osteophyte

A summary of ratings for all 4 assessors is included in Table 2. All four assessors identified the presence of anterior femoral neck osteophytes in the majority of cases (range 69–86%). After exclusion of Rater #2 as an outlier, a mean of 82% (78-86%) of cases were found to have osteophytes in this location. A significant proportion of patients (mean 27%, range 23–31%) were found to have large or extensive osteophytes there.

### 3.3. Inter-Rater Reliability (IRR)

Inter-rater reliability was calculated manually in Apple Numbers [version 5.3 (5989)]. With all 4 assessors included, inter-rater reliability was calculated to be 68%. After exclusion of a single divergent assessor, IRR exceeded 70%.

## 4. Discussion

This study identified the presence of anterior femoral neck osteophyte in the large majority of this Australian cohort of THA patients. This finding was unanimous among all raters who assessed the preoperative plans. This finding is not surprising, given the experience of the authors and the inclusion of periarticular osteophyte formation as one of the hallmark radiographic features of osteoarthritis. If not addressed at the time of surgery, the presence of osteophytes at the anterior aspect of the femoral neck may significantly increase the risk of anterior bony impingement and subsequent posterior instability.

The ability to visualize osteophytes and assess the risk of impingement intraoperatively maybe depends on the choice of surgical approach. In particular, osteophytes at the anterior femoral neck may be more difficult to visualize during cases done via the posterior approach to the hip, when compared to the direct anterior, anterolateral, or direct lateral approaches. In its 2015 annual report, the UK National Joint Registry reported the use of the posterior approach in 62% of all primary total hip arthroplasty cases [14]. Given the commonality of both the posterior approach and anterior femoral neck osteophytes, a significant risk of unaddressed osteophytes remains. Numerous factors may be considered in the selection of a surgical approach, and surgeons may be hesitant to alter their approach in order to facilitate access to anterior osteophytes. Surgeons who utilize the posterior approach may elect to focus specific attention on assessing for and resecting anterior osteophytes during femoral preparation.

The impact of unaddressed anterior neck osteophytes likely also depends on a given patient's pattern of pelvic motion. It has been estimated that 52% of patients experience increased anterior pelvic tilt when sitting compared to standing [15]. These patients would be at particularly high risk of posterior instability in flexion and internal rotation due to the presence of anterior femoral neck osteophytes. A further analysis of this study cohort reveals a similar pattern, in that approximately half of the patients in each group exhibit anterior pelvic tilt with sitting (no osteophyte: 46%; minor: 58%; extensive: 50%) [unpublished data].

One limitation of this study is the utilization of an Australian cohort. This population may not be reflective of other global populations. There were no patient exclusions during analysis of a consecutive series, however, so this cohort is likely to represent the true local population. In addition, the images included in the surgical plans were generated for the purpose of implant positions and sizing and were not optimized for assessment of osteophytes. The relatively low resolution may limit the accuracy of this assessment and may have contributed to the moderate inter-rater reliability. The outlying rater was the most junior member of the research team. Differences in training may have contributed to variability among assessors.

## 5. Conclusions

A large majority of total hip arthroplasty patients may be expected to exhibit osteophyte formation at the anterior aspect of the femoral neck. Approximately 27% were found to exhibit large or extensive osteophytes in this area. These pathoanatomic variations must be incorporated into surgical planning and addressed intraoperatively in order to mitigate the risk of postoperative impingement and instability. Patients who experience anterior pelvic tilt with sitting will be at particularly high risk. During procedures performed via the posterior approach, focused effort and attention may be required to visualize and resect anterior femoral neck osteophytes. Planned future directions include incorporation of an impingement and instability model into preoperative surgical planning for THA.

## Figures and Tables

**Table 1:** Descriptive statistics of patient cohort.

**Patient Parameter**	**N = 413**
Age (years) [mean, range]	63 (32-91)

Gender [n, %]	
Male	216 (52%)
Female	197 (48%)

Laterality [n, %]	
Right	227 (55%)
Left	186 (45%)

**Table 2:** Summary of ratings for presence of anterior femoral neck osteophyte, by assessor. Analysis was repeated with Rater #2 excluded due to divergence from the others.

**Assessor**	**Absent**	**Minor**	**Extensive**	**Any osteophyte (minor + extensive)**
	**[n, **%** of cases]**	**[n, **%** of cases]**	**[n, **%** of cases]**	**[n, **%** of cases]**
#1	56 (14%)	230 (56%)	127 (31%)	357 (86%)
**#2**	**128 (31**%**)**	**224 (54**%**)**	**61 (15**%**)**	**285 (69**%**)**
#3	75 (18%)	243 (59%)	95 (23%)	338 (82%)
#4	89 (22%)	210 (51%)	113 (27%)	323 (78%)

## Data Availability

The data used to support the findings of this study are available from the corresponding author upon request.
